# High Percentage of Isomeric Human MicroRNA and Their Analytical Challenges

**DOI:** 10.3390/ncrna2040013

**Published:** 2016-12-02

**Authors:** Joseph N. Mwangi, Norman H. L. Chiu

**Affiliations:** Department of Chemistry and Biochemistry, Joint School of Nanoscience and Nanoengineering, University of North Carolina at Greensboro, Greensboro, NC 27412, USA; joseph.njoroge.mwangi@gmail.com

**Keywords:** microRNA, nucleotide composition, isomers

## Abstract

MicroRNA (miR) are short non-coding RNAs known to post-transcriptionally regulate gene expression, and have been reported as biomarkers for various diseases. miR have also been served as potential drug targets. The identity, functions and detection of a specific miR are determined by its RNA sequence, whose composition is made up of only 4 canonical ribonucleotides. Hence, among over two thousand human miR, their nucleotide compositions are expected to be similar but the extent of similarity has not been reported. In this study, the sequences of mature human miR were downloaded from miRBase, and collated using different tools to determine and compare their nucleotide compositions and sequences. 55% of all human miR were found to be structural isomers. The structural isomers of miR (SimiR) are defined as having the same size and identical nucleotide composition. A number of SimiR were also found to have high sequence similarities. To investigate the extent of SimiR in biological samples, three disease models were chosen, and disease-associated miR were identified from miR2Disease. Among the disease models, as high as 73% of miR were found to be SimiR. This report provides the missing information about human miR and highlights the challenges on the detection of SimiR.

## 1. Introduction

In comparison to the structures of other biopolymers that exist in living cells, ribonucleic acid (RNA) has a smaller set of monomeric units, which consist of four ribonucleotides, namely adenosine (A), uridine (U), guanosine (G), and cytidine (C). Furthermore, two of the nucleobases are purine (A and G) and the other two are pyrimidine (U and C). Despite of this rather simple RNA structure, the biological functions of RNA have continued to grow. In order to create a variety of RNA functions, single-stranded RNA molecules rely on the Watson-Crick base pairing and the intramolecular interactions with the hydroxyl group at the 2′ position of each ribonucleotide to generate relatively stable RNA folding [1,2]. To overcome the limitation on having only four canonical ribonucleotides, RNA may undergo over 100 different types of RNA modifications, which in turn may induce unique RNA structures and/or functions. In general, RNAs are categorized by their functions. For example, messenger RNA are templates for protein synthesis, whereas transfer RNA convert the genetic codes into their corresponding amino acid residues during the protein synthesis [3]. Since the discovery of small non-coding microRNA (miR), more than 2000 human miR have been identified [4,5,6,7]. Specific miR can post-transcriptionally regulate gene expression by binding directly with messenger RNA, which results in either blocking the biosynthesis of corresponding protein or cleaving the messenger RNA with the assistance from a protein called Dicer [8,9,10,11]. In the former case, the binding between a specific miR and its messenger RNA target does not require 100% complementary base matching. This binding mechanism, therefore, allows the same miR to regulate more than one specific gene expression. Collectively, miR are estimated to regulate as much as 60% of gene expression in our bodies. Many specific miR have been associated to various diseases [12,13,14,15,16,17,18]. In some cases, several different miR are reported to be associated with the same disease. Besides serving as diagnostic or prognosis biomarkers, some miR have also been recognized as potential drug targets [19,20,21,22,23,24]. To further explore the potentials of miR in medical related studies, accurate detection of a specific miR is critical. All the current analytical methods for miR detection [25,26,27,28,29,30,31] rely on the ability to distinguish a particular RNA structure, which may include its size, nucleotide composition and/or RNA sequence. For determining the size of a specific RNA molecule, the conventional or chip-based gel electrophoresis methods are commonly used [32,33]. Alternatively, sequencing methods can be used to determine the size of an RNA molecule as well as its RNA sequence, the latter information is particularly important to the identification of a specific RNA molecule including miR [34,35]. Until the recent development on the technology for next generation sequencing, the use of complementary nucleic acid probe(s) to detect specific RNA target has been the preferred method to achieve fast turnaround time and multiplexing for both RNA identification and quantitation [36]. To address the specificity issue of probe-based methods, mass spectrometric methods for measuring unmodified and modified RNA have been developed [37,38]. Although the structural information on human miR has been available for some time, no study on comparing their structural similarities has been reported yet. In this report, we determine the extent of structural similarity among all the human mature miR; and discuss its consequence to the detection of miR.

## 2. Results and Discussion

### 2.1. Size Distribution of Human Mature microRNA

**Figure 1:**
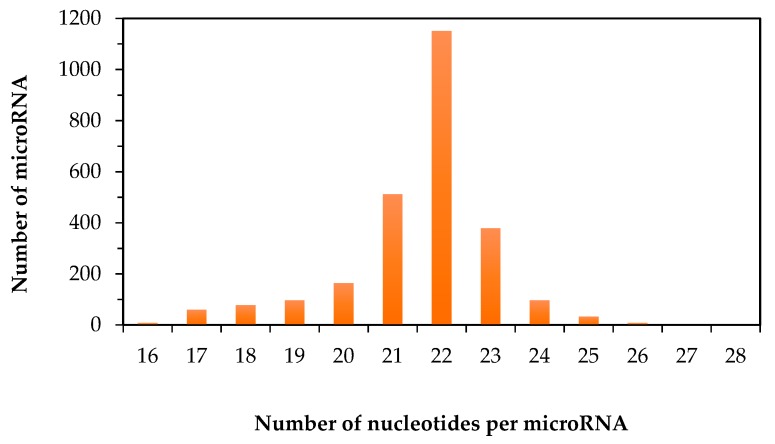
Distribution of human mature microRNA as a function on the number of nucleotides per microRNA.

In the literature, the reported values for the minimum and maximum size of human mature miR often vary. Besides the possible errors in the earlier reports, this discrepancy could also be due to the ever-expanding list of human miR. Based on the information that was available from miRBase on 19 August 2016, an attempt to determine the correct minimum and maximum size of human mature miR was carried out. There are in total 2588 human miR. The size of the human mature miR ranges from 16 to 28 nucleotides (Figure 1). This makes the average size of mature human miR to be 22 nucleotides. As shown in Figure 1, among all the human miR, 44% of them have the same size that equals to the average size of 22 nucleotides. Only 16% of mature human miR have the size of either smaller than or equal to 20 nucleotides. The rest of the human miR are longer than 22 nucleotides. The size distribution of human miR explains why the probe-based methods, in which a complementary DNA oligo or an analogue is used as a molecule probe to recognize a specific miR, is a viable approach for the detection of human miR. If an RNA molecule has 22 nucleotides, it theoretically creates 4^22^ or over seventeen trillion possibilities for its RNA sequence. In other words, the RNA sequence of each human miR is unique in the entire human transcriptome, thus the recognition of a specific miR by matching its RNA sequence with a complementary DNA probe should provide sufficient specificity. However, in practice, non-specific binding of DNA probe is often unavoidable, especially when the size and the annealing position of the complementary DNA probe are fixed by the actual size of miR. If non-specific binding is not eliminated, it will lead to false-positive results on the detection of miR. To address this issue, different ways to improve the specificity of probe-based methods have been reported [39,40]. However, similar to many other analytical measurements, the outcome from using any specific method to detect miR will partly depend on the sample complexity. To the best of our knowledge, there is currently no specific method for isolating only miR from a biological sample. The closest purification method that is available for miR research can only remove RNA longer than 200 nucleotides from a total RNA sample [27]. Hence, transfer RNA and other types of RNA smaller than 200 nucleotides may co-exist with human miR in the same sample. In order to achieve high specificity and accuracy on the detection of miR, the information on the similarity of the properties of human miR can be very useful. For this reason, the rest of this report focuses on comparing the nucleotide compositions and RNA sequences of human miR.

### 2.2. Isomeric microRNA

**Figure 2:**
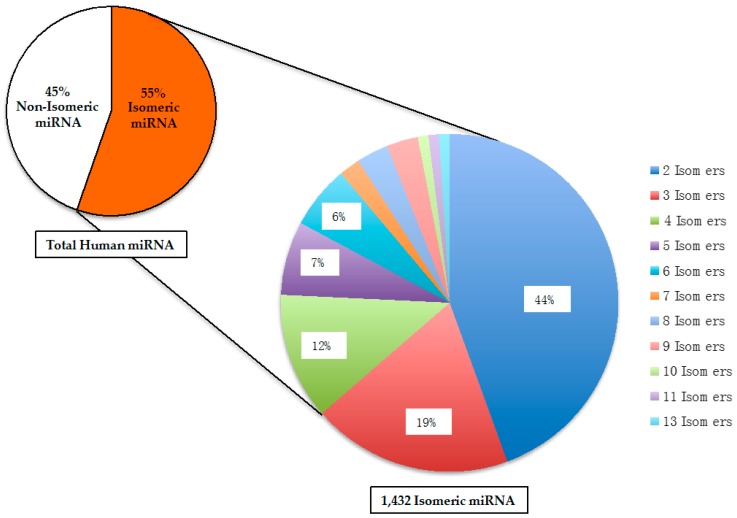
Distribution of isomeric and non-isomeric human mature microRNA. Isomeric microRNA are defined as having identical nucleotide composition, but different RNA sequences. In total, there are 2588 human microRNA.

If two different RNA molecules have the same size and identical nucleotide composition, they would be chemically defined as structural isomers. The structural isomers of miR is hereby referred as “SimiR”. It is important to note that an acronym called isomiR has been reported in the literature, and does not refer to isomeric miR [41]. Among all the human mature miR, 1432 (or 55%) of them are SimiR. As shown in Figure 2, the isomeric human miR can be further categorized by the number of isomers, which have the same size and identical nucleotide composition.

Among the groups of isomeric miR, the highest number of isomers is 13, and only one group of 13 isomers exists (Table 1). The majority of isomeric human miR (44%) belong to the group of 2 isomers. In total, there are 315 different pairs of isomeric miR. Similar to the other types of RNA molecules, the modifications of miR have been reported, which include both adenylation and uridylation [42,43]. The results of those RNA modifications would change the RNA sequence of miR, which may then alter the extent of miR isomerism. However, due to the lack of information on those RNA modifications in the entire collection of human miR, it is beyond the scope of this report to determine and compare the various extents of miR isomerism with or without any RNA modifications.

### 2.3. Disease-Associated Isomeric microRNA

**Figure 3:**
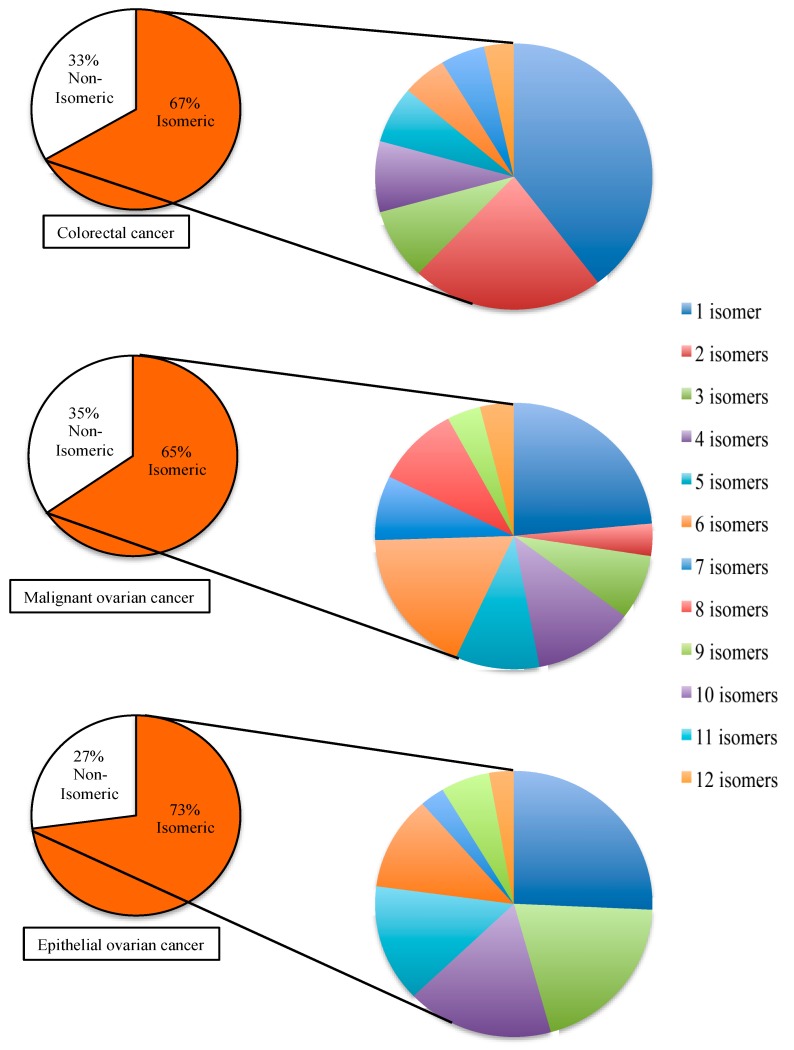
Isomerism of microRNA in selected disease models. The disease-associated microRNAs are categorized by the selected diseases. In each disease model, a specific disease-associated microRNA is defined as isomeric microRNA if its nucleotide composition is identical to another human microRNA. The percentages of isomeric disease-associated microRNA are shown in the smaller pie charts on left hand side. The total number of disease-associated microRNA in each case is different—colorectal cancer has 87 microRNA, malignant ovarian cancer has 78 microRNA, and epithelial ovarian cancer has 48 microRNA. For each selected disease, the isomeric disease-associated microRNA are further subcategorized by the number of isomers that co-exist among the human microRNA and the percentage distributions of each group of isomers are shown in the pie charts on the right hand side.

Through many studies, a lot of human miR have been associated to diseases. In some cases, more than one specific miR is associated to the same disease. For this reason, whether the disease-associated miR would co-exist in the same biological sample or not, there are needs to measure as many disease-associated miR as possible. For instance, a particular cellular response can be monitored more accurately by measuring a small panel of miR that are associated to the same disease. To evaluate the implication of miR isomerism to the detection of disease-associated miR, three different disease models were selected. The three selected disease models are colorectal cancer, malignant ovarian cancer, and epithelial ovarian cancer. Based on the information available from miR2disease database, the total number of disease-associated miR in each selected model is different from each other. In comparison to the entire collection of human miR, the distribution of isomeric miR in each selected disease model is shown in Figure 3. In the case of colorectal cancer, there are 87 miR associated to the disease, and 67% of them are isomeric. In the case of malignant ovarian cancer, there are 78 miR associated to the disease and 65% of them are isomeric to other human miR. In the case of epithelial ovarian cancer, there are 48 miR associated to the disease and 73% of them are isomeric. In comparison to the 55% of isomeric miR among all the human miR, the percentage of isomeric miR in the selected disease models are significantly higher.

### 2.4. Sequence Similarities among Isomeric microRNA

The minimum difference between the RNA sequences of two isomeric miR is two nucleotides. In Table 1, the sequences of six different pairs of isomeric miR were found to be different by only two nucleotides. Although some of their names are very similar, the isomeric miR with high sequence similarity in Table 2 are encoded by different genes. With the high sequence similarities, these isomeric miR are expected to target the same messenger RNA. Hence, it is important to accurately determine which particular isomer is responsible for a specific post-transcriptional regulation of gene expression.

### 2.5. Analytical Challenges from Isomeric microRNA

As summarized in Table 3, the current analytical methods for detecting miR can be divided into three categories. In terms of their analytical performance, each category has its pros and cons for the detection of a specific miR (Table 3). Theoretically, the applications of these analytical methods to differentiate isomeric miR are feasible but the outcome may vary. Firstly, there is no doubt that the current sequencing methods including the next generation sequencing technology can differentiate isomeric miR even with high sequence similarities. However, in comparison to the other analytical methods, the next generation sequencing technology has the highest cost on the consumable materials and the highest demand on data analysis. For those analytical methods whose endpoint measurement involves measuring the mass, the molecular mass of isomeric miR are exactly identical, thus mass spectrometry with high mass resolution will not be useful for the differentiation of isomeric miR. Recently Biba and his co-workers [44] completed an in-depth study on using different chromatographic methods to resolve small isomeric RNA molecules with high sequence similarities. Their experimental results indicated that there are high probabilities for small isomeric RNAs to be co-eluted from a chromatographic column. Hence, the coupling of liquid chromatography to mass spectrometry may not provide sufficient resolving power to differentiate isomeric miR. Since 100% sequence coverage is not always achievable in tandem mass spectrometry of RNA and the mass difference between uridine and cytidine is only 1 Da, it remains challenging to re-sequence RNA with ≥22 nucleotides by using tandem mass spectrometry. Hence, there are limitations on using mass spectrometric methods to accurately identify or differentiate isomeric miR in a single sample. By optimizing the annealing conditions, the probe-based methods should have the ability to distinguish two or limited number of isomeric miR, providing the differences in the RNA sequence of isomeric miR are not at or near the 5′ or 3′ termini. Also, the size and the annealing position of the DNA probe or its analogue are fixed by the actual size of miR.

## 3. Materials and Methods

In this study, the RNA sequence of mature human miR and the location of their corresponding gene in the human genome were downloaded from the latest version of miRBase (miRBase 21) database (http://www.mirbase.org) [45] on 19 August 2015. The online Mongo oligo mass calculator v2.06 [46] was used to calculate the nucleotide composition of each miR. In-house Excel-based tools were used to determine the size of miR and identify isomeric human miR. For the analysis of sequence similarity, MAFFT [47], a multiple sequence alignment tool that is freely accessible online, was employed to generate the data according to the guidelines for the program.

The three models of disease used in this study were randomly chosen based on the information available from the miR2Disease database, which is a collaboration between Indiana University School of Medicine and the Harbin Institute of Technology [48]. Before analyzing the disease-associated miR data as described above in this section, the information of each individual miR was crosschecked with information in the miRBase database.

## 4. Conclusions

Based on the results of this study, the size of a human mature miR ranges from 16 to 28 nucleotides, which is slightly different from the earlier reports. Among 2588 mature human miR, 44% have the same size with 22 nucleotides. Despite of this bias in their size distribution, and the limitation of only four canonical ribonucleotides, each specific miR still has its own unique RNA sequence among the other RNA molecules in the human transcriptome with the exceptions on gene duplication. However, for the first time, we report that high percentage (55%) of human mature miR are isomeric. MicroRNA that have the same size and identical nucleotide composition are defined as structural isomers of miR and hereby referred as SimiR. In reference to the whole collection of human miR, SimiR have been identified among miR that have been associated with a specific disease. Although each SimiR has a unique RNA sequence, some SimiR have relative high sequence similarities. Since all the current analytical methods for the detection of miR rely on the ability to distinguish a particular RNA structure, SimiR poses a new analytical challenge to the current methodologies.

## Figures and Tables

**Table 1:** The largest group of structural isomers among all the human microRNA, and their individual gene location. Three of the structural isomers listed below have identical RNA sequence, which could be the results of gene duplication.

miRNA	Sequence (5′–3′)	Chromosome	Location
miR-21-5p	UAGCUUAUCAGACUGAUGUUGA	17	NC_000017.11 (59841266..59841337)
miR-95-3p	UUCAACGGGUAUUUAUUGAGCA	4	NC_000004.12 (8005301..8005381)
miR-100-3p	CAAGCUUGUAUCUAUAGGUAUG	11	NC_000011.10 (122152229..122152308)
miR-513b-5p	UUCACAAGGAGGUGUCAUUUAU	X	NC_000023.11 (147199044..147199127)
miR-519a-3p	AAAGUGCAUCCUUUUAGAGUGU	19	NC_000019.10 (53752397..53752481)
miR-519b-3p	AAAGUGCAUCCUUUUAGAGGUU	19	NC_000019.10 (53695213..53695293)
miR-522-3p	AAAAUGGUUCCCUUUAGAGUGU	19	NC_000019.10 (53751211..53751297)
miR-548am-5p	AAAAGUAAUUGCGGUUUUUGCC	X	NC_000023.11 (16627012..16627085)
miR-548c-5p	AAAAGUAAUUGCGGUUUUUGCC	12	NC_000012.12 (64622509..64622605)
miR-548h-5p	AAAAGUAAUCGCGGUUUUUGUC	6	NC_000006.12 (131792172..131792231)
miR-548k	AAAAGUACUUGCGGAUUUUGCU	11	NC_000011.10 (70283955..70284070)
miR-548o-5p	AAAAGUAAUUGCGGUUUUUGCC	20	NC_000020.11 (38516563..38516632)
miR-4789-5p	GUAUACACCUGAUAUGUGUAUG	3	NC_000003.12 (175369540..175369621)

**Table 2:** Examples of isomeric human microRNA that have high sequence similarities, and their gene location within the human genome.

miRNA	Sequence (5′–3′)	Chromosome	Location
let-7a-2-3p	CUGUACAGCCUCCUAGCUUUCC	11	NC_000011.10 (122146522..12214659)
let-7e-3p	CUAUACGGCCUCCUAGCUUUCC	19	NC_000019.10 (51692786..51692864)
miR-301b-3p	CAGUGCAAUGAUAUUGUCAAAGC	22	NC_000022.11 (21652981..21653058)
miR-301a-3p	CAGUGCAAUAGUAUUGUCAAAGC	17	NC_000017.11 (59151136..59151221)
miR-378a-3p	ACUGGACUUGGAGUCAGAAGGC	5	NC_000005.10 (149732825..14973289)
miR-422a	ACUGGACUUAGGGUCAGAAGGC	15	NC_000015.10 (63870930..63871019)
miR-20b-5p	CAAAGUGCUCAUAGUGCAGGUAG	X	NC_000023.11 (134169809..13416987)
miR-17-5p	CAAAGUGCUUACAGUGCAGGUAG	13	NC_000013.11 (91350605..91350688)
miR-148a-3p	UCAGUGCACUACAGAACUUUGU	7	NC_000007.14 (25949919..25949986)
miR-148b-3p	UCAGUGCAUCACAGAACUUUGU	12	NC_000012.12 (54337216..54337314)

**Table 3:** Current categories of analytical methods for measuring microRNA.

Analytical Method	Specificity for Detecting Specific microRNA	Sample Throughput	Costs	Differentiation of Isomeric microRNA
By Sequencing	★★★	Low to High	$$–$$$	Yes
By Mass Measurement	★★★	Medium	$$	Size- and Sequence-dependent
By Complementary Probe	★★ to ★★★	Low to High	$$	Sequence-dependent
